# Possible Luttinger liquid behavior of edge transport in monolayer transition metal dichalcogenide crystals

**DOI:** 10.1038/s41467-020-14383-0

**Published:** 2020-01-31

**Authors:** Guanhua Yang, Yan Shao, Jiebin Niu, Xiaolei Ma, Congyan Lu, Wei Wei, Xichen Chuai, Jiawei Wang, Jingchen Cao, Hao Huang, Guangwei Xu, Xuewen Shi, Zhuoyu Ji, Nianduan Lu, Di Geng, Jing Qi, Yun Cao, Zhongliu Liu, Liwei Liu, Yuan Huang, Lei Liao, Weiqi Dang, Zhengwei Zhang, Yuan Liu, Xidong Duan, Jiezhi Chen, Zhiqiang Fan, Xiangwei Jiang, Yeliang Wang, Ling Li, Hong-Jun Gao, Xiangfeng Duan, Ming Liu

**Affiliations:** 1 Key Laboratory of Microelectronic Devices & Integrated Technology, Institute of Microelectronics, Chinese Academy of Sciences, 100029 Beijing, China; 2 Institute of Physics, Chinese Academy of Sciences, 100190 Beijing, China; 3 School of Information and Electronics, MIIT Key Laboratory for Low-Dimensional Quantum Structure and Devices, Beijing Institute of Technology, 100081 Beijing, China; 4 School of Information Science and Engineering, Shandong University, 250100 Jinan, China; 5 Institute of Semiconductors, Chinese Academy of Sciences, 100083 Beijing, China; 6 Department of Physics and Key Laboratory of Artificial Micro- and Nano-structures of Ministry of Education, Wuhan University, 430072 Wuhan, China; 7 State Key Laboratory for Chemo/Biosensing and Chemometrics, College of Chemistry and Chemical Engineering, Hunan University, 410082 Changsha, China; 8 Department of Chemistry and Biochemistry and California Nanosystems Institute, University of California, Los Angeles, CA 90095 USA

**Keywords:** Electronic properties and materials, Electronic devices, Two-dimensional materials

## Abstract

In atomically-thin two-dimensional (2D) semiconductors, the nonuniformity in current flow due to its edge states may alter and even dictate the charge transport properties of the entire device. However, the influence of the edge states on electrical transport in 2D materials has not been sufficiently explored to date. Here, we systematically quantify the edge state contribution to electrical transport in monolayer MoS_2_/WSe_2_ field-effect transistors, revealing that the charge transport at low temperature is dominated by the edge conduction with the nonlinear behavior. The metallic edge states are revealed by scanning probe microscopy, scanning Kelvin probe force microscopy and first-principle calculations. Further analyses demonstrate that the edge-state dominated nonlinear transport shows a universal power-law scaling relationship with both temperature and bias voltage, which can be well explained by the 1D Luttinger liquid theory. These findings demonstrate the Luttinger liquid behavior in 2D materials and offer important insights into designing 2D electronics.

## Abstract (web summary)

Though metallic edge states in monolayer transition metal dichalcogenide have been observed before, how these states contribute to charge transport remains unclear. Here, the authors quantify the edge state contribution to electrical transport in monolayer MoS_2_/WSe_2_ field-effect transistors, revealing a dominated non-linear edge transport at low temperature, indicating possible Luttinger liquid behavior.

## Introduction

Surface states of bulk three-dimensional (3D) semiconductors are critical factors to be considered in designing and fabricating electronic devices, as they can result in trapping and scattering, or acting as recombination centers for free carriers^1^. An in-depth understanding of the surface states has enabled scientists and engineers to optimize the performance of integrated circuits during the past a few decades. Analogous to the surface states of bulk crystals, the edge termination and reconstruction in monolayer two-dimensional (2D) materials (e.g., molybdenum disulfide (MoS_2_) and graphene) can lead to the edge electronic states^2–5^, which bring remarkable properties to these 2D materials and spark considerable intriguing applications^6^. The metallic electronic states at the edges of 2D transition metal dichalcogenides (TMD) have been predicted by first-principle calculations^7,8^. However, experimental investigations of the 2D edge states are scarce and little is known about the effects as well as the fundamental mechanism of edge states on electronic transport and performance of monolayer TMD-based electronic devices, which are critical for evaluating their performance for future nanoelectronic devices.

Herein we report a systematic investigation of the edge contribution to charge transport property in monolayer MoS_2_ and WSe_2_ field-effect transistors (FETs) and discovery of edge state-induced nonlinear electronic transport and underlying mechanism. Scanning probe microscopy (SPM), scanning Kelvin probe force microscopy (SKPFM) and first-principle calculations reveal that the edge states dominate the charge transport and are responsible for this nonlinear behavior. We further find that the current–voltage characteristics of charge transport show a power-law scaling with respect to both temperature and bias voltage, which is consistent with Luttinger liquid theory and suggests that the edge states are one-dimensional metal with dominant electron–electron interactions.

## Results

### Edge states conductance quantization

**Fig. 1:**
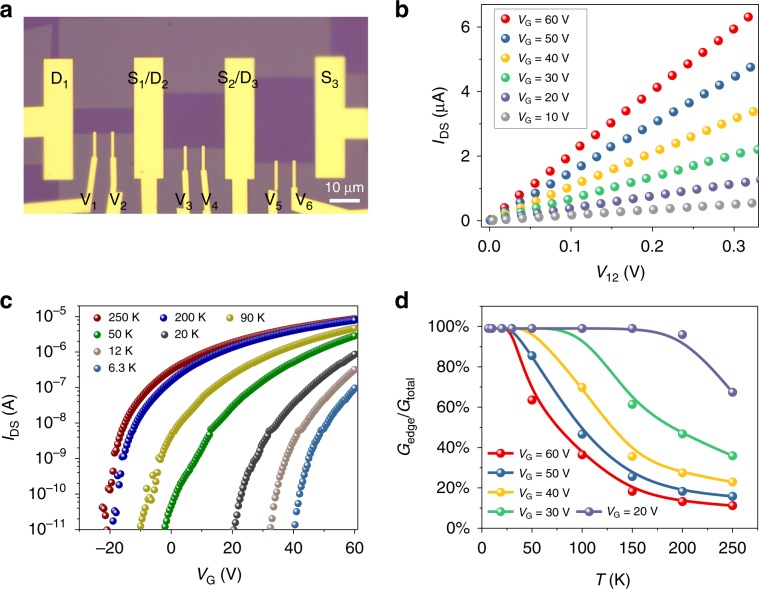
Edge states conductance quantification. **a** Optical image of monolayer MoS_2_ FETs with inner probes (V_1_–V_6_) to sense voltage drop within channel. The scale bar is 10 μm. **b** Output ($I_{{{\mathrm{D}}_{1}}{\mathrm{S}}_{1}}$–$V_{{\mathrm{12}}}$) curves of monolayer MoS_2_ FET at 250 K with gate voltages 10 to 60 V. **c** The transfer curves ($I_{{{\mathrm{D}}_{1}}{{\mathrm{S}}_{1}}}$–$V_{{\mathrm{G}}}$) in MoS_2_ device measured at $V_{{{\mathrm{D}}_{1}}{{\mathrm{S}}_{1}}}=1$ V from 250 to 6.3 K. **d** The ratios of edge conductance (*G*_edge_) to total conductance (*G*_total_) over large temperature and gate voltage range based on the experimental measurements as described in the Supplementary Note 1.

To quantitatively evaluate the contribution of charge transport along the edge states at different gate voltages and temperatures, we fabricated multi-probe device from one monolayer MoS_2_ flake (Fig. 1a). This method can eliminate contributions from contact^9–11^. Figure 1b shows the output characteristic curves ($I_{{{\mathrm{D}}_{1}} {{\mathrm{S}}_{1}}}$–$V_{12}\,{\mathrm{curves}}$) of the monolayer MoS_2_ device under gate voltage from 60 to 10 V. The transfer curves ($I_{{{\mathrm{D}}_{1}}{{\mathrm{S}}_{1}}}$–$V_{{\mathrm{G}}}$) at $V_{{{\mathrm{D}}_{1}}{{\mathrm{S}}_{1}}} = 1\; {\mathrm{V}}$ bias with different temperature are shown in Fig. 1c. More importantly, the conductance contributed from the edge and bulk can be separated by measuring and quantifying the characteristic curves (*I*–*V*) curves of the devices with different edge lengths and bulk area (Supplemental Note 1). Notably, experimental data from 6.3 to 350 K reveal the edge conductance ratios (*G*_edge_/*G*_total_) increase with decreasing temperature, and become larger than 50% at the temperature below 50 K (Fig. 1d), which indicates that the edge transport plays a dominant role over the bulk transport at low temperature. This higher contribution from edge transport at lower temperature may be attributed to the carrier freezing out in bulk semiconducting MoS_2_. With increasing gate voltage, more carriers populate at the semiconducting MoS_2_ bulk channel, leading to higher bulk conductance and lower edge/total conductance ratio.

To demonstrate the generality of one-dimensional transport behavior in TMDs FETs, a typical p-type monolayer tungsten diselenide (WSe_2_) FET with hole carrier is studied. It demonstrates a similar trend, with the edge conductance dominating the overall transport at lower temperature regime (Supplementary Fig. 2). Thus, these systematical studies reveal the monolayer TMD exhibit a similar edge state characteristic regardless of the carrier type.

### STM/S measurements

**Fig. 2:**
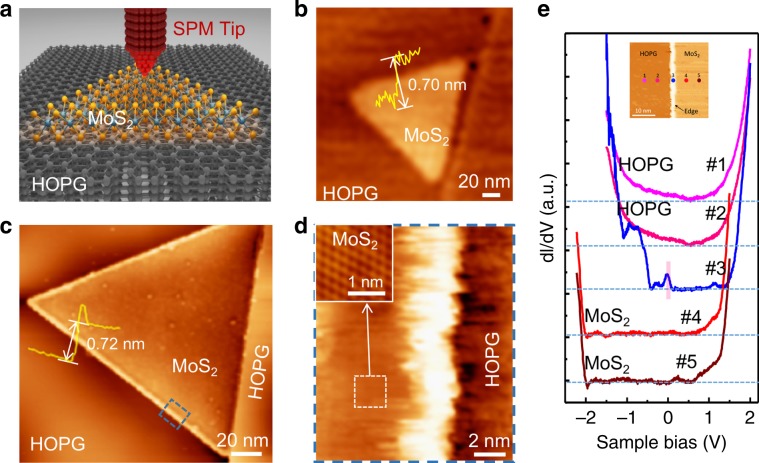
SPM characterization of monolayer MoS_2_ islands on HOPG. **a** Schematic of SPM measurements process. **b** AFM image of a typical triangular MoS_2_ island, showing no obvious brightness differences between the edge and the bulk. The profile line (yellow) shows an apparent height of 0.70 nm of the triangular flake, corresponding to the single-layer MoS_2_ feature. **c** STM image of the triangular MoS_2_ island grown alongside the edge of the substrate terrace, clearly showing the electronic edge states with a brim of very high conductance along the island edges. The profile line (yellow) also shows a significant protrusion at the edge. The apparent height of the triangular flake is 0.72 nm, consistent with the AFM measurement in **b**. **d** Zoomed-in STM image of the area indicated by the dark-blue rectangle in **c**. Inset: atomic resolution image of MoS_2_ obtained from the area indicated by the white dashed square. **e** d*I*/d*V* spectra taken across the MoS_2_ edge from HOPG to the bulk MoS_2_. The color of the curves is consisted with the color of marked positions in the inset. The tunneling spectrum (#3) acquired at the MoS_2_ edge shows clearly electronic states (marked by pink) around Fermi level, revealing its metallic feature. STM scanning parameters: *U* = −1.0 V, *I* = 100 pA (**c**) and (**d**) *U* = −1.0 V, *I* = 800 pA for the inset.

To further probe the nature of edge charge transport, high-resolution scanning probe techniques are used to reveal both the topography and the electronic property of monolayer MoS_2_ crystal (Fig. 2a). The atomic-thin MoS_2_ is grown on highly oriented pyrolytic graphite (HOPG) conductive substrate, which is good for scanning tunneling microscope and spectroscopy (STM/S) measurements. Figure 2b shows a typical AFM image of a triangular MoS_2_ island. The apparent height of the island is illustrated by the yellow profile line and is nearly 0.70 nm, which is consistent with the single-layer MoS_2_ feature. It is notable that no obvious height differences can be found between the edge and the bulk of monolayer MoS_2_ crystal in the AFM image, which is similar to former studies of MoS_2_ on HOPG surface^12,13^. Figure 2c shows a typical STM image of one triangular MoS_2_ island obtained in an ultrahigh-vacuum chamber (base pressure < 3 × 10^−10^ mbar). The profile line of the MoS_2_ island shows its apparent height of 0.72 nm, also confirming the monolayer nature of the MoS_2_ sample. Notably, the STM image reveals a brim with high brightness contrast around the edge of MoS_2_ island (Fig. 2c), in contrast to AFM image with a largely flat edge topography.

To get more detailed structural and electronic information about the edges, we zoomed in the area near the edge of the MoS_2_ island marked by the blue dashed-line in Fig. 2c and obtained the image as shown in Fig. 2d. This image presents a significant brightness contrast along the MoS_2_ edge, which is recognized as the metallic edge states by a brim of very high conductance extending all the way along the edges, consistent with previous STM/STS measurements^8,12,14^. An atomic resolution STM image reveals the clean MoS_2_ surface with clear atomic structure (as shown in the inset of Fig. 2d). These AFM and STM studies together demonstrate the atomically clean topography of the MoS_2_ island and that the bright brim observed in the STM images is not due to different edge topography, but due to the conductive electronic states at the edge. Metallic edge states of the MoS_2_ crystal are also confirmed by scanning tunneling spectroscopy (STS) measurements. Figure 2e shows d*I*/d*V* spectra taken across the MoS_2_ edge from HOPG to the bulk MoS_2_ (data-recording positions are shown in the inset). The tunneling spectrum (#3) acquired at the MoS_2_ edge shows clearly electronic states (marked by pink) around Fermi level compared to spectra (#4 & #5) from MoS_2_ bulk, revealing its metallic feature. The edge states stay consistently at different positions along the MoS_2_ edge, as shown in the Supplementary Fig. 3. Differently, the spectra from HOPG (#1 & #2) show metallic and the spectra from MoS_2_ (#4 & #5) show semiconductor feature.

### First-principle calculations

To further understand the origin of the edge electronic states, we conducted first-principle calculations based on density-functional-theory (DFT) and non-equilibrium Green’s function (NEGF) by using the Perdew-Burke-Ernzerhof (PBE) version of the generalized gradient approximation (GGA)^15–17^. The calculated band structure indicates that the appearance of additional bands within bandgap (highlighted by the red line in the Supplementary Fig. 4a), which can be attributed to broken crystalline symmetry and the presence of dangling bonds at the edge. These additional bands cross the Fermi level and are localized at the edge atoms as shown by the calculated charge density distribution (Supplementary Fig. 4b), confirming that the metallic states are associated with the edge atoms.

**Fig. 3:**
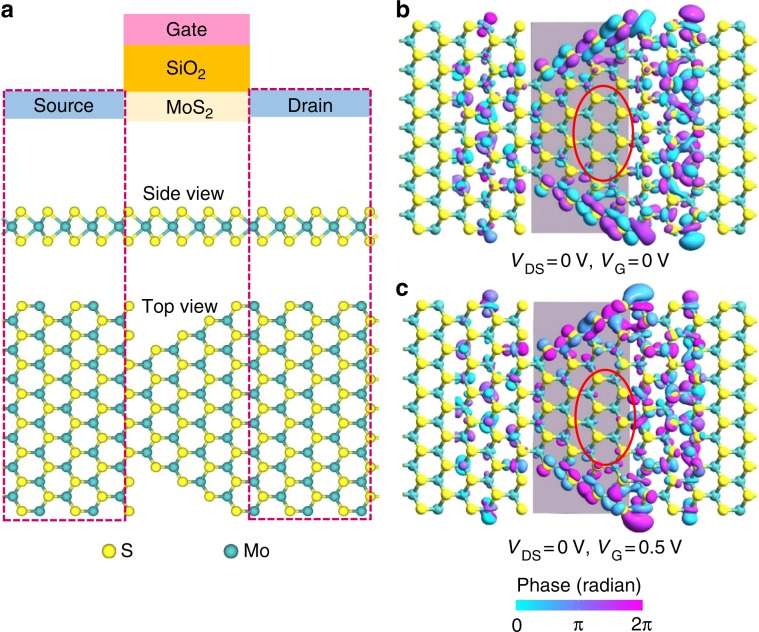
Calculations of charge transport on edge state of monolayer MoS_2_. **a** Schematic of monolayer MoS_2_ FET (Uppermost row); side view (Middle row) and top view (Lowest row) of monolayer MoS_2_. The isosurface plots for transmission eigenstates of the MoS_2_ channel at the k-point Γ(0,0) and the energy of 0.05 eV under the conditions of *V*_DS_ = 0 V and *V*_G_ = 0 V in **b**, and *V*_DS_ = 0 V and *V*_G_ = 0.5 V in **c**. The evolving of the carriers in bulk is revealed as highlighted by the red ellipse in **b** and **c**, although only a small gate voltage (0.5 V) is applied in simulation.

We have further simulated the electrical charge transport behavior across the triangular MoS_2_ domain by Atomistix Tool Kit (ATK)^15–17^ by constructing a two-probe model comprised of a semiconducting MoS_2_ sample with source and drain ends, in which the source and drain are defined by the highly doped (10^14^ e/cm^2^) n-type MoS_2_ to reduce extra calculation time (Fig. 3a). Such MoS_2_-based device is simulated under different bias voltage conditions (Fig. 3b, c). Figure 3b shows the transmission eigenstates spread along the edge of monolayer MoS_2_, indicating that the electrons tend to transport along the edge of MoS_2_ (*V*_G_ = 0 V and *V*_DS_ = 0 V). As the back-gate voltage increases from 0 to 0.5 V, a few more transmission eigenstates start to locate at the bulk of MoS_2_ channel (Fig. 3c), indicating that more electrons populate at the MoS_2_ bulk, leading to an increase in bulk conductance and a decrease of the relative contribution from the edge conductance contribution, which is consistent with the experimental observations as shown in Fig. 1.

### SKPFM measurement

**Fig. 4:**
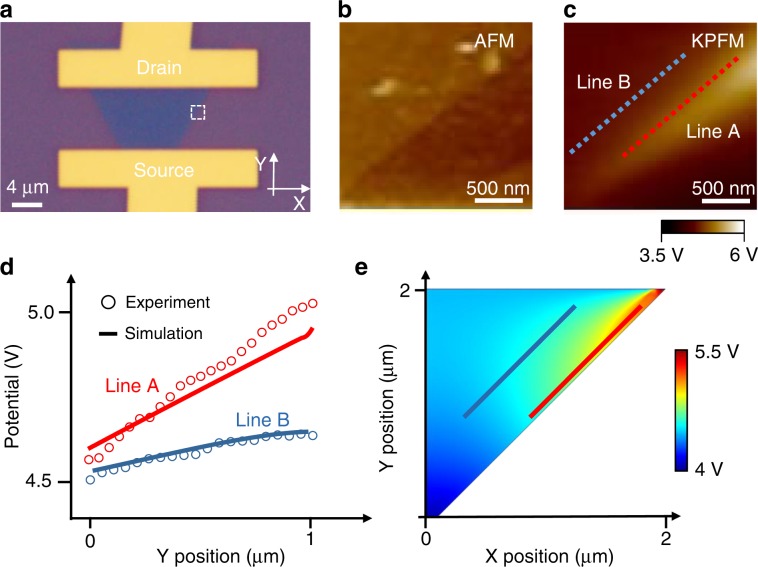
SKPFM measurement of monolayer MoS_2_ FET. **a** Optical image of the MoS_2_ FET device. The white rectangle region is the one selected for SKPFM measurement. **b** AFM image of the selected region. **c** Local potential map of the selected region at the bias voltage of *V*_DS_ = 8 V and *V*_G_ = 0 V. Line scans of the potential both across the edge (Line A) and the bulk (Line B) of the MoS_2_ flake. **d** Voltage potential comparison between the experimental data and simulation data. The experimental potential difference at the edge is 524 mV (Circles in Line A) and the difference at the bulk is 137 mV (Circles in Line B). The significant high potential at the edge indicates that charges flow at edge under the given bias voltage. The simulation results (Solid line) match well with the experimental results. **e** Simulated potential mapping of MoS_2_ channel based on the finite-element method, consistent with the experimental mapping in **c**.

To directly reveal the role of the 1D metallic edge state in monolayer MoS_2_ device, we have further mapped the local electrical potential of the monolayer MoS_2_ FET through the SKPFM measurements^18,19^, which is an AFM-based method that can record the local potential variation of a sample surface, relative to a metallic probe. Using this method, local potential variation, resulting from edge states, and bulk states in the MoS_2_ sample, can be directly imaged under working conditions, and thus the electronic transport path can be visualized. The optical image of the channel region of the monolayer MoS_2_ device is depicted in Fig. 4a. The AFM image depicted from the white rectangle in Fig. 4a is shown in Fig. 4b, and its corresponding SKPFM potential mapping is shown in Fig. 4c with working conditions (*V*_DS_ = 8 V, and *V*_G_ = 0 V). Two typical potential curves (as depicted by Line A and Line B across the edge and the bulk of the triangle-shaped MoS_2_ sample, respectively) are shown in Fig. 4d. We can see that significant potential contrast is exhibited along the edge (depicted by Line A) and the bulk (Line B). The potential difference along the edge, about 524 mV, is much larger than the one along bulk, 137 mV (in equal perpendicular distance). These results clearly reveal that the electrons prone to flow along the edge of the MoS_2_ sample under the given bias voltage. Furthermore, the potential cross the MoS_2_ channel is simulated by the finite-element method as shown in Fig. 4e, which is consistent with the experimental observation in Fig. 4c. Additionally, this observations agree well with the previous results measured by microwave impedance microscopy, which shows that the electron charge is prone to flow at the edge by measuring the local conductance contrast in edge and bulk material^20^.

### Nonlinear charge transport

**Fig. 5:**
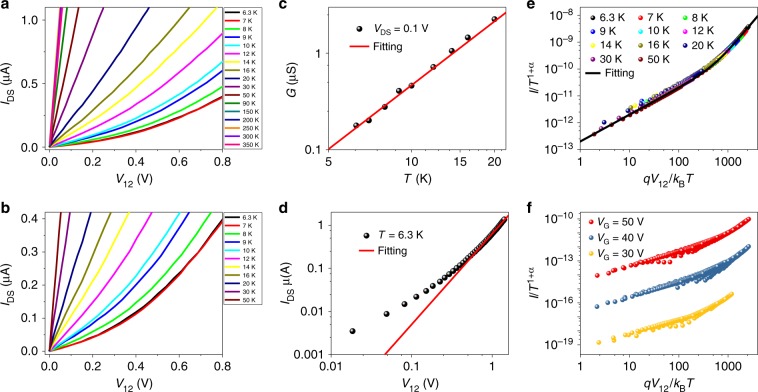
Evidence of Luttinger liquid transport in monolayer MoS_2_ FET. **a** Typical (*I*_DS_–*V*_12_) curves measured under *V*_G_ = 60 V, varying temperature from 350 to 6.3 K. **b** The (*I*_DS_–*V*_12_) below 50 K are shown, illustrating clear nonlinear charge transport behavior in monolayer MoS_2_ FET. **c** Power law relation of conductance *G* with temperature (*G* ∼ *T*^α^) at low bias *V*_12_ = 0.1 V, obtaining a fitting exponent *α* = 2.2 from the red fitting curve. **d** Power law relation of the current with the voltage $I_{{\mathrm{DS}}}\sim V_{{\mathrm{12}}}^\beta$ at low temperature *T* = 6.3 K, having a fitting exponent *β* = 2.2. **e** The output transport data and the corresponding curve plotted by *I*_DS_/*T*^1+α^ against *qV*_12_/*k*_B_*T* under the gate voltage of *V*_G_ = 60 V. The nonlinear (*I*_DS_–*V*_12_) data scale well onto a universal curve over the temperature range from 50 to 6.3 K. The fitting curve indicates nonlinear Luttinger liquid behavior. **f** (*I*_DS_–*V*_12_) data scale onto the universal curve replotted as *I*_DS_/T^1+α^ versus *qV*_12_/*k*_B_*T* under other gate biases: *V*_G_ = 30, 40, and 50 V, respectively.

We have further investigated the temperature dependence of the electrical transport in monolayer MoS_2_ FET device. Figure 5a shows the output (*I*_DS_–*V*_12_) curves of the device at large temperature range from 350 to 6.3 K, where the channel current decreases rapidly with decreasing temperature *T*. The linear (*I*_DS_–*V*_12_) characteristics are retained above 50 K, indicating an ohmic behavior. With further decreasing temperature, the characteristic (*I*_DS_–*V*_12_) curves show clear nonlinear behavior above the threshold voltages (*V*_T_)^11,21^ (Fig. 5b). A possible mechanism of the nonlinear power-law dependence of (*I*_DS_–*V*_12_) characteristics is due to Coulomb blockade (CB)^22^. However, this theory requires isolated conductive dots separated by a very thin tunneling layer, and the threshold voltage *V*_T_ extracted in CB model depends linearly on temperature^23^. These two characteristics of CB model do not agree with our results, suggesting a new mechanism of nonlinear transport in our devices (Supplementary Note 6).

Another theory used for nonlinear charge transport is Luttinger liquid model. In this theory, the tunneling amplitude of an electron into an Luttinger liquid vanishes as a power–law relation with the energy of the tunneling electron^24–26^, which results in a power–law dependence of conductance G with temperature by formula of (*G* = *I*/*V* ∝ *T*^*α*^) and the current with voltage by *I* ∝ *V*^*β*^, in which *α* and *β* are the tunneling exponents. In our experimental measurements, the conductance *G* = *I*_DS_/*V*_12_ shows a power–law behavior with temperature as *G* ∼ *T*^*α*^ and gives *α* = 2.2 at *V*_12_ = 0.1 V (Fig. 5c). Similarly, at *T* = 6.3 K, $I_{{\mathrm{DS}}}\sim V_{{\mathrm{12}}}^\beta$ shown in Fig. 5d gives *β* = 2.2. These experimental results are consistent with the prerequisite of the Luttinger liquid model.

An additional feature of the Luttinger liquid theory is that the universal scaling curve of all (*I*_DS_–*V*_12_) curves at different temperatures can be obtained when *I*_DS_/*T*^1+α^ is plotted against *eV*_12_/*k*_B_*T* as^27–29^:

1 $$I_{{\mathrm{DS}}} = I_0T^{{\mathrm{1 + }}\alpha } \cdot {\mathrm{sinh}}(\gamma eV_{{\mathrm{12}}}/k_{\mathrm{B}}T)\left| {\Gamma \left(\frac{{1 + \beta }}{2}{\mathrm{ + }}i\gamma \frac{{eV_{{\mathrm{12}}}}}{{\pi k_{\mathrm{B}}T}}\right)} \right|^2,$$

where Γ(*x*) is the gamma function, *k*_B_ is the Boltzmann constant, *α* and *β* are the tunneling exponents as mentioned above, *I*_0_ is the fitting parameter and *γ*^−1^ denotes the number of tunnel barriers in the transport path of Luttinger liquid.

According to Eq. (1), we plot *I*_DS_/*T*^1+*α*^ against *eV*_12_/*k*_B_*T* with our experimental results (Fig. 5e). The nonlinear (*I*_DS_–*V*_12_) curves scale well onto a universal curve over the temperature range from 50 to 6.3 K. This universal curve can be well fitted to Eq. (1), as shown by the solid curve in Fig. 5e, in which the parameters’ values *α* = 2.2, *β* = 2.2, and *γ*^−1^ = 200 are derived. These extracted data of *α* and *β* are identical to the values from the power exponent of *G* *∼* *T*^*α*^ (Fig. 5c) and $I_{{\mathrm{DS}}}\sim V_{{\mathrm{12}}}^\beta$ (Fig. 5d) as obtained above, respectively. In Fig. 5f, the output data are plotted as *I*_DS_/*T*^1+*α*^ versus *eV*_12_/*k*_B_*T* at various gate voltages (*V*_G_ = 30, 40, 50 V). Under these different gate voltages, the fitting parameter *γ*^−1^ based on Eq. (1), is again constantly equal to 200. The parameter *γ*^−1^ is related to the number of tunneling barriers that lies along the transport path and determines a crossover from ohmic behavior to a power–law dependence in the Luttinger liquid^27,28^. Here, the constant *γ*^−1^ under different gate voltages further validates Luttinger liquid model. For monolayer WSe_2_ FET, the output curves (*I*_DS_–*V*_12_) at low temperature shows nonlinear behavior and the corresponding curves can also be scaled to a universal curve with *α* = 2.15, *β* = 3, and *γ*^−1^ = 100 according to the Eq. (1) (Supplementary Fig. 7).

This parameter *γ*^−1^ is further discussed in the Supplementary Note 8 with MoS_2_ devices with various channel lengths (edge lengths). In these devices, the cross points obtained by fitting output data as *I*_DS_/*T*^1+*α*^ against *eV*_12_/*k*_B_*T* are analyzed, and the linear dependence of *γ*^−1^ as function of edge length is again obtained in the Supplementary Fig. 8, in which the number of tunneling barriers (*γ*^−1^) increase with increasing channel lengths (edge lengths).

Based on the different *α* values extracted from MoS_2_ and WSe_2_ FETs, the electron interaction (MoS_2_) or hole interaction (WSe_2_) parameter *g* can be calculated to be 0.102 and 0.106, respectively (Supplementary Note 9). The parameters from all MoS_2_ and WSe_2_ devices are summarized in the Supplementary Table 1 in Supplemental Information. All these data here demonstrates that the Luttinger parameter *g* *≪* 1, as often discussed in Luttinger model^24,27,30,31^, indicating a strong repulsive electron–electron (hole–hole) interactions along 1D charge transport path in edge-state-dominated MoS_2_ (WSe_2_) crystals and this result can be generally extended to all semiconducting TMD crystals.

## Discussion

These findings raise interesting implications on the generality of the Luttinger model for monolayer MoS_2_ transistors. We note that the agreement with Eq. (1) has been advocated as a clear evidence for tunneling into 1D or quasi-1D Luttinger metallic liquid^28,32^. Thus, direct evidences from both theory and experiment suggest that the edge states are 1D Luttinger metallic liquid in 2D crystal, which accounts for the nonlinear charge transport at low temperature. A schematic process of edge-state-dominated 1D metallic charge transport in atomic-thin 2D crystal based on Luttinger metallic liquid model is shown in Supplementary Fig. 11. It shows that electron transport from one Luttinger liquid to another Luttinger liquid through 1D tunneling across possible defects or impurities along the metallic edges in monolayer 2D materials, as shown above by the example of MoS_2_ and WSe_2_ crystals.

We have witnessed two previous works demonstrate that the metallic mirror-twin boundaries in MoS_2_^33^ and MoSe_2_^34^ show Luttinger liquid behavior, in which the Luttinger parameter *g* are extracted to be 0.2 for MoS_2_^33^ and 0.5 for MoSe_2_^34^, respectively. These values are much larger than the parameter *g* (0.11–0.08) extracted in our experiment from edge states, indicating that stronger electron–electron (or hole–hole) repulsive interactions along the edges of TMD than the one at the grain boundaries of TMD. In addition, a more general argument could be strengthened that metallic line defects (edges or grain boundaries) in 2D semiconductors all show Luttinger liquid behavior and are ideal 1D systems.

Peierls transition have seen observed in many 1D metallic systems^34,35^, in which the periodic lattice deformation, resulting in a sudden increase in sample resistance at the transition temperature. However, all the temperature-dependency resistances of our samples in the Supplementary Fig. 12 do not show sudden transition as same as ref. ^34^. Thus, we believed that the likelihood of Peierls transition is low in metallic edge states of TMD (Supplementary Note 12).

Together, our studies demonstrate for the first time that the metallic edge states in 2D TMD materials behave like a 1D Luttinger liquid, which leads to nonlinear transport at smaller gate voltages and lower temperature. Importantly, the electron–electron interaction in such edge states in 2D-TMDs may be fine-tuned by the gate voltage, offering a new interesting model system for probing 1D quantum transport. The apparent metallic edge states may also lead to important implications for 2D-TMD devices. For example, the metallic edge state may suggest a possible pathway to engineer proper edge contact to 2D materials and facilitate charge injection^36^.

## Methods

### Device fabrication

Monolayer MoS_2_ (WSe_2_) were exfoliated from the commercial MoS_2_ (WSe_2_) crystals (SPI Supplies) by scotch-tape method on Si/SiO_2_ substrate. The source and drain contacts (10 nm Ti/70 nm Au for MoS_2_ and 70 nm Au for WSe_2_) were formed through electron beam lithography (EBL), electron beam evaporation and then a lift-off process. The rectangular MoS_2_ (WSe_2_) channels were patterned by a second EBL step and subsequently etched using O_2_/SF_6_ plasma.

### Electrical measurements

Electrical measurements were conducted by using a Keithley 4200 semiconductor parameter analyzer and a Lakeshore probe station. The multi-probe measurement configuration is also shown in Fig. 1a. The inner probes (*V*_1_ ~ *V*_6_) sense voltage drop. A bias voltage (*V*_DS_) is applied across the drain and source terminals. The channel current *I*_DS_ is measured at the end of drain terminal. The voltage drops measured in this configuration can exclude any effects related to contacts, in which the voltage difference (*V*_12_ = *V*_1_ − *V*_2_, *V*_34_ = *V*_3_ − *V*_4_, and *V*_56_ = *V*_5_ − *V*_6_) is measured between the two inner terminals.

### Scanning tunneling microscopy/spectroscopy

STM measurements were carried out in a custom-built multi-chamber ultra-high vacuum system. The base pressure was better than 3 × 10^−10^ mbar. Before STM measurements, the sample was annealed at 150 °C for over 12 h to remove possible adsorbates. A chemically etched tungsten tip was used for imaging. The STM images were recorded in constant current mode with tunneling current in the range 100–800 pA. The MoS_2_/HOPG sample was transferred to the ultrahigh-vacuum chamber and degassed at 150 °C for 12 h before STM analyses. STS measurements were using standard lock-in techniques with a voltage modulation of 10 mV and frequency of 973 Hz. Before real-sample STS measurements, the tip was calibrated on a clean Au(111).

### First-principles calculations

The electronic transport properties are carried out by ATK toolkit on the basis of density-functional theory in combination with the nonequilibrium Green’s function^15,16^. The local density approximation (LDA) and the wave function is expanded by the single-zeta plus polarization basis for all atoms^17^. The MoS_2_ device configuration and a cut-plane representation of the transmission eigenstates throughout the device central region under different gate voltages are shown in Fig. 3. In calculations, the parameters are set as follows: The k-point sampling is 1, 1, and 100 in the *A*, *B*, and *C* directions, respectively. The real space grid techniques are used with the energy cutoff of 150 Ry in numerical integrations. The quantum mechanical transmission probability of electrons T(*E,V*) can be given as^16,17^:

$${\mathrm{T}}\left( {E,V} \right) = {\mathrm{Tr}}[{\mathrm{\Gamma }}_{\mathrm{L}}(E,V)G^{\mathrm{R}}(E,V){\mathrm{\Gamma }}_{\mathrm{L}}(E,V)G^{\mathrm{A}}(E,V)],$$

where *E*, *V* are the energy and applied voltage, respectively, Γ_L/R_ is the contact broadening functions related with the left/right electrode and *G*^R/A^ is the retarded/advanced Green’s function of the central region.

First-principles calculations for edge states based on density functional theory (DFT) in Part S5 of Supplementary Information were conducted using the Perdew-Burke-Ernzerhof (PBE) version of the generalized gradient approximation (GGA).

## Supplementary information

Supplementary Information

## Data Availability

All data needed to evaluate the conclusions in the paper are present in the paper and/or the Supplementary Materials. Additional data related to this paper may be requested from the authors.
