# Supplementary material for: Possible Luttinger liquid behavior of edge transport in monolayer transition metal dichalcogenide crystals
Source: Nat Commun. 2020 Jan 31;11:659. doi: 10.1038/s41467-020-14383-0 (PMC6994668; doi:10.1038/s41467-020-14383-0)
Supplement: Supplementary file 1 — Supplementary Information [file 41467_2020_14383_MOESM1_ESM.pdf]

Supplementary Information

Possible Luttinger Liquid Behavior of Edge Transport in Monolayer Transition Metal Dichalcogenide Crystals

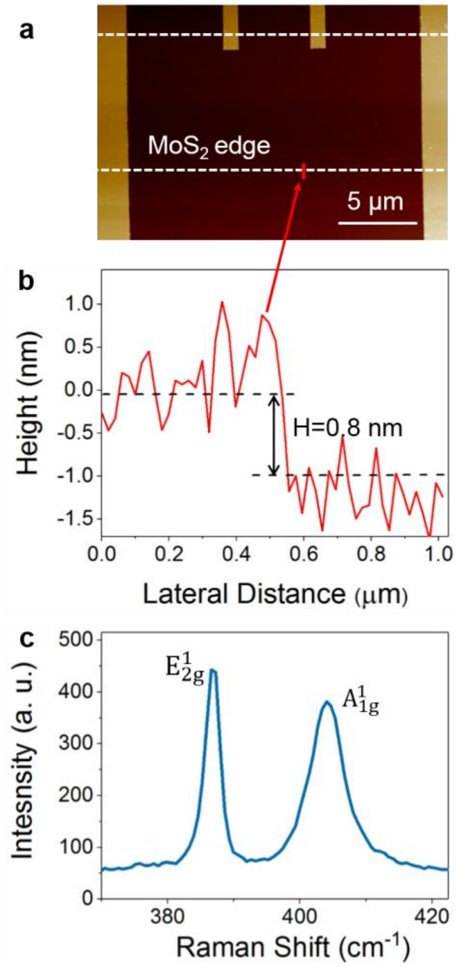

Supplementary Figure 1. Characterization of monolayer MoS_2. **a**, AFM image of a sheet of MoS_2 on SiO_2/Si with Multi-probe metal contact. The white dotted line marks the position of the edge of MoS_2 flake. The scale bar is 5 μm. **b**, Cross-sectional plot along the red line in (a). Height was measured to be around 0.8 nm, which confirms the MoS_2 sample is monolayer in thickness. **c**, The separation between vibration modes of the in-plane peak (E^1_{2g}) and out-of-plane peak (A^1_{1g}) was found to be 18.5 cm^{-1}, confirming that the sample is a single-layer.

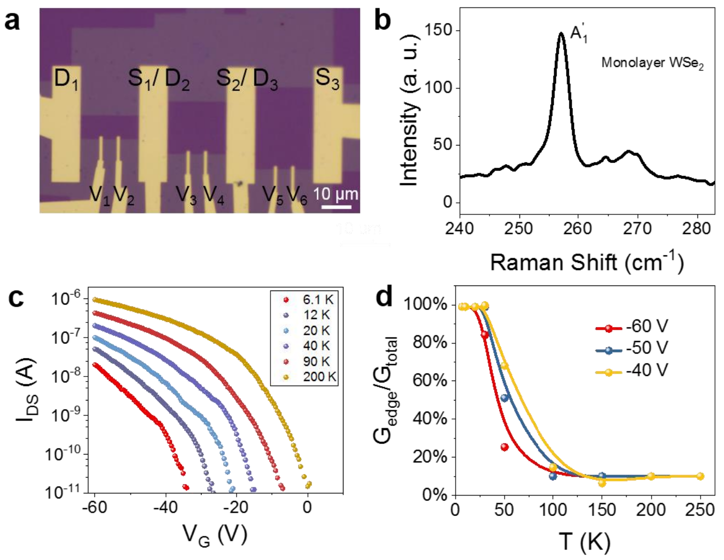

Supplementary Figure 2. Monolayer WSe_2 FET. **a**, Optical image of monolayer WSe_2 FETs. The scale bar is 10 μm. **b**, Raman spectra of monolayer WSe_2. **c**, Transfer curves $(I_{\mathrm{D_1S_1}} - V_{\mathrm{G}})$ of monolayer WSe_2 FET at $V_{\mathrm{D_1S_1}} = 1$ V bias with temperature varying from 6.1 K to 200 K. **d**, Ratio of edge conductance to total conductance over large temperature range under gate voltage of -60 V, -50 V and -40 V.

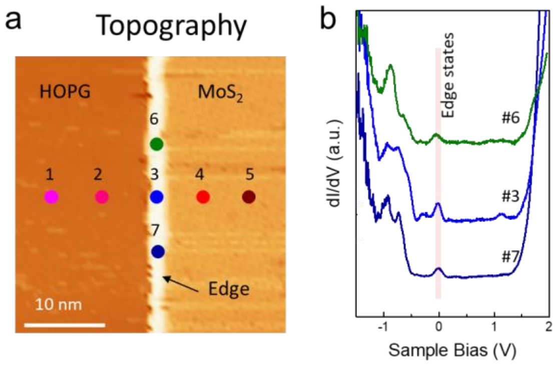

Supplementary Figure 3. STS measurements of the MoS_2 island edge. a, STM topography image of monolayer MoS_2 island on HOPG substrate, demonstrating an obvious bright brim at the edge (V=-2.0 V, I= 0.5 nA). **b**, STS spectra taken along the MoS_2 edge, clearly demonstrating the consistent metallic edge states. The spectra numbers were corresponding to the positions marked in **a**. Edge states are clearly shown in Spectra #3, #6, and #7. Spectra #1- #5 have been shown in the Fig.2e in the main manuscript.

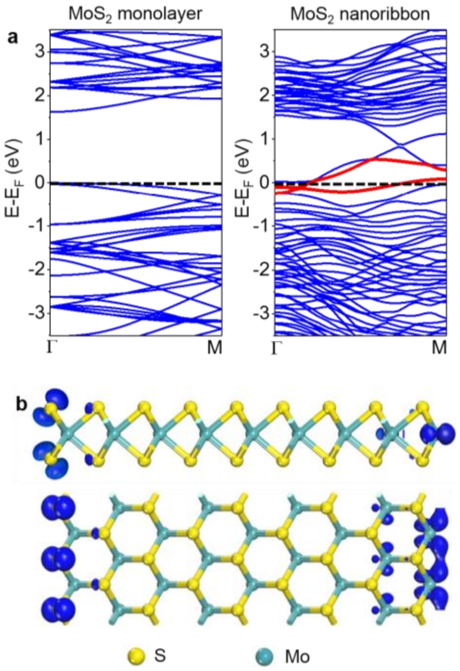

Supplementary Figure 4. DFT calculations of monolayer MoS_2 edge states. a, Calculated band structures of the monolayer MoS_2 (left) and a 2.1-nm-wide MoS_2 nanoribbon (right). The horizontal black lines represent the Fermi level. The additional energy bands highlighted by the red line cross the Fermi level indicate metallic states in the MoS_2 nanoribbon, besides the normal energy bands (the blue lines). **b**, Side view (upper row) and top view (lower row) of the electron wave functions. The charge density of additional metallic electronic states is localized mainly at the edge atoms, confirming that the metallic states are associated with the edge atoms.

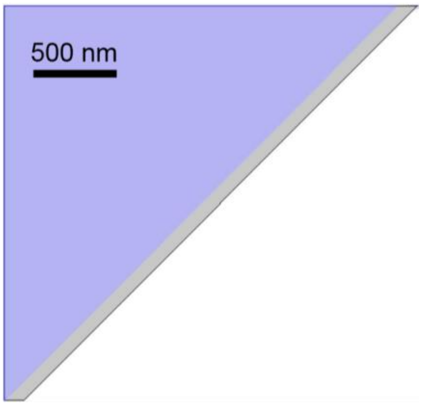

Supplementary Figure 5. The schematic of the 2D finite-element analysis for MoS_2 with edge states. The sample contains two parts: one is bulk (highlighted by the violet) and another is edge (highlighted by the grey) as shown in the Supplementary Figure 5. The width of edge used in this simulation is 100 nm. The conductivity of the bulk part and edge part is 0.1 S/m and 10000 S/m based on the Reference [1].

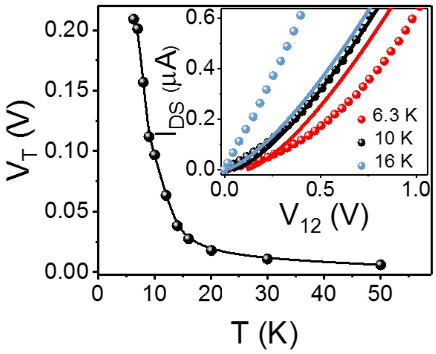

Supplementary Figure 6. The fitting of nonlinear behavior using Coulomb blockade model. The threshold voltage (V_T) decreases nonlinearly with increasing temperature; The dots in the inset are *I*-*V* data measured at temperatures of 16, 10 and 6.3 K, respectively. The colored solid lines are fitting curves. The fitting curves at 16 and 6.3 K has a clear shift away from the experiment data, indicating the *I*-*V* data cannot be explained by a Coulomb blockade model. The measurements are taken under gate voltage of 60 V.

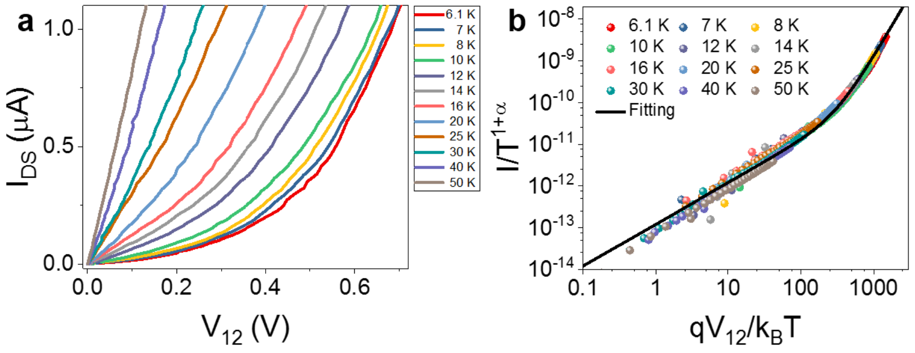

Supplementary Figure 7. The scaling of the current-voltage characteristics of monolayer WSe_2 FETs. **a**, Typical $(I_{\mathrm{DS}} - V_{12})$ curve measured under $V_{\mathrm{G}} = -60\,V$ with varying temperature from 50 K to 6.1 K, demonstrating clear nonlinear charge transport behavior in monolayer WSe_2 FET. With lowering temperature, I-V become more pronounced nonlinear. **b**, $(I_{\mathrm{DS}} - V_{12})$ data scale onto the universal curve replotted as $I_{DS}/T^{1+\alpha}$ versus qV_{12}/k_BT under gate voltage $V_{\mathrm{G}} = -60\,V$.

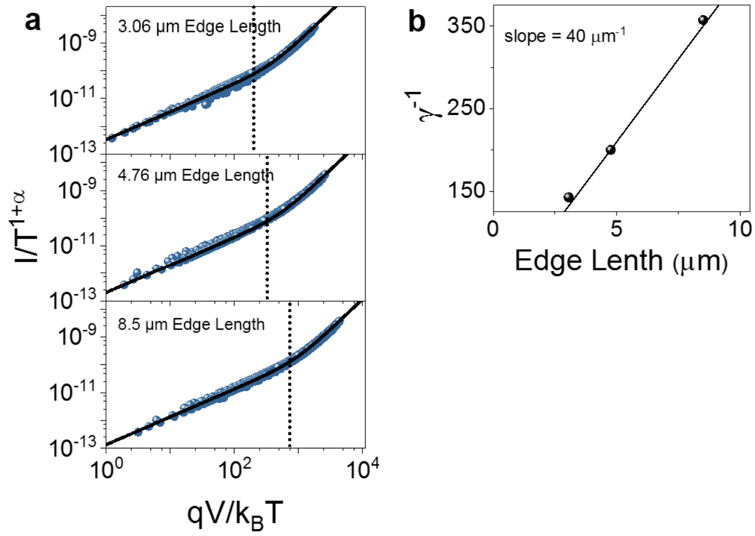

Supplementary Figure 8. The scaling of the current-voltage characteristics of monolayer MoS_2 FETs. **a**, Scaled source-drain current $I/T^{1+\alpha}$, as functions of $qV/k_B T$ of monolayer MoS_2 FETs, with different channel length (L_{edge}). The solid lines are fits to equation (1) as described in the main text. The black dashed lines act as eye guides to for the position of the crossover in the curves. **b**, Relation of the extracted parameter γ^{-1} with different edge lengths in MoS_2 FETs. γ^{-1} depends linearly on the length of edge.

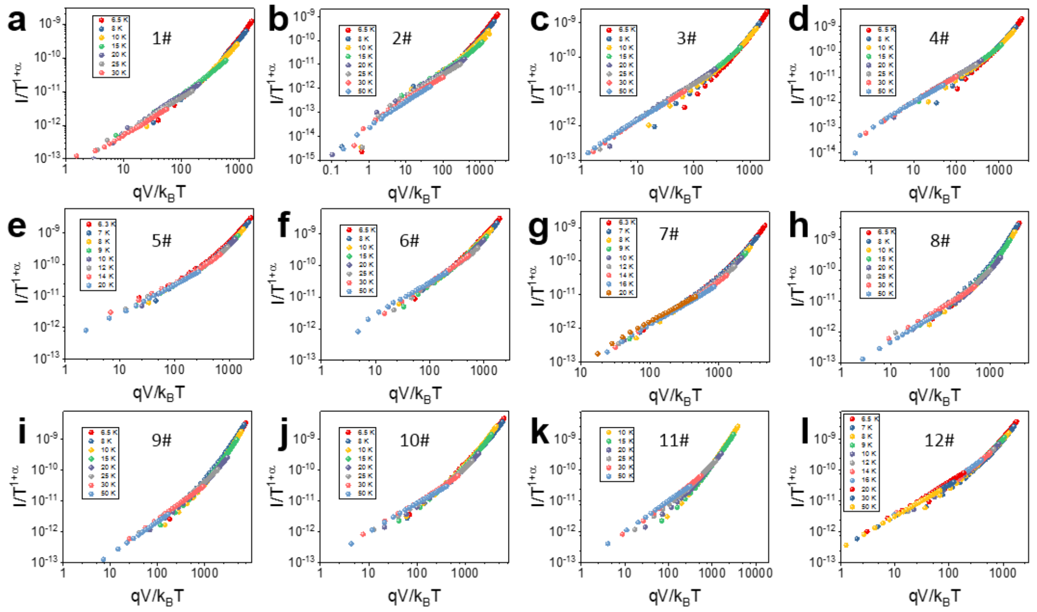

Supplementary Figure 9. Luttinger liquid behavior in 12 monolayer MoS_2 devices.

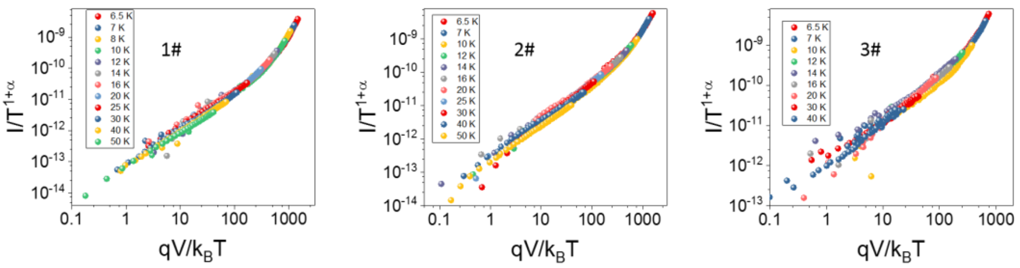

Supplementary Figure 10. Luttinger liquid behavior in monolayer WSe_2 devices.

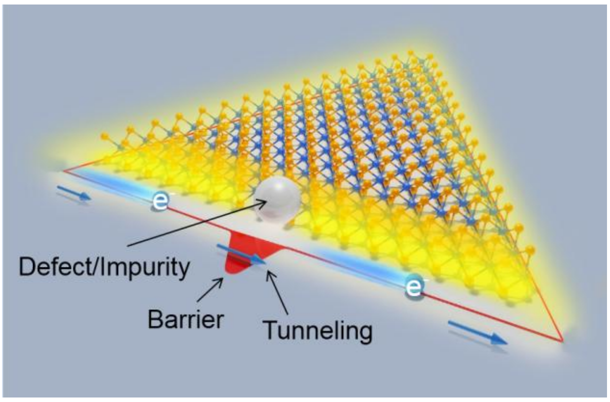

Supplementary Figure 11. Schematic of Luttinger-liquid-based charge transport on monolayer MoS_2 edge states. Electrons transport along the metallic edge of the monolayer MoS_2 flake as highlighted by the yellow, tunneling defects or impurities (represented by the white sphere) in MoS_2 crystal.

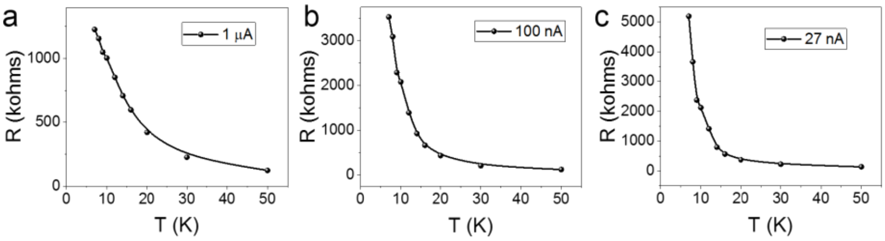

Supplementary Figure 12. Temperature dependent resistance of monolayer MoS_2 as applied constant current of (a) 1 µA, (b) 100 nA and (c) 27 nA.

sample	MoS_2												WSe_2		
#	1	2	3	4	5	6	7	8	9	10	11	12	1	2	3
α	2.8	2.8	2.4	2.4	2.3	2.3	3.1	2.4	2.4	2.5	2.5	2.2	2.1	2	2
g	0.08	0.08	0.09	0.09	0.10	0.10	0.07	0.09	0.09	0.09	0.09	0.10	0.106	0.11	0.11

Supplementary Table 1. Measured power-law exponent α and Luttinger parameter g from each MoS_2/WSe_2 devices.

Supplementary Note 1. Edge state conductance quantification

The edge states conductance can be measured by using the device in Figure 1a in the main text, in which the device MoS_2 is fabricated with inner probes (V_1~V_6) to sense the voltage drop in channel. The influence of contact resistance can be eliminated in this method. The conductance measured between inner probes can be expressed by the sum of the conductance from two parts, that is, from the edge and the bulk, respectively. We describe that the conductivity at the MoS_2 edge is $G_{0,edge}$ and the conductivity at the MoS_2 bulk is $G_{0,bulk}$, respectively. The width of edge is 3 nm based on the result of scanning tunneling microscopy in Figure 2d in main text. Thus, we have the conductance between terminals V_1 and V_2 and between terminals V_3 and V_4 in the following equations:

$$G_{12} = G_{0,edge} \times W_{edge}/L_{12} + G_{0,bulk} \times W_{12}/L_{12}, \quad (1)$$

$$G_{34} = G_{0,edge} \times W_{edge}/L_{34} + G_{0,bulk} \times W_{34}/L_{34}, \quad (2)$$

in which G_{12} and G_{34} are the conductance measured between probe V_1 and V_2, between probe V_3 and V_4, respectively. L_{12} and L_{34} are the channel length between probe V_1 and V_2, between probe V_3 and V_4. W_{edge} is the edge width.

Thus, the conductance between probe V_1 and V_2 contributed by the edge and bulk can be expressed in the following:

$$G_{12,edge} = G_{0,edge} \times W_{edge}/L_{12,edge} \quad (3)$$

$$G_{12,bulk} = G_{0,bulk} \times W_{12}/L_{12} \quad (4)$$

The contribution by the edge conductance over the total conductance between two probes can be evaluated by the ratio: $G_{12,edge}/G_{12}$.

So based on the method stated above, we summarize the conductance at the edge G_{edge}, the total conductance between two probes G_{total} and the ratio G_{edge}/G_{total} under various temperatures and gate voltages in the Fig. 1d in the main text.

Supplementary Note 2. MoS_2 sample characterization by AFM and Raman

Supplementary Figures 1a and 1b present the characterization of the MoS_2 sample by AFM imaging. The apparent height is 0.8 nm, which confirms that the MoS_2 sample is monolayer in thickness. Supplementary Figure 1c shows the characterization of monolayer MoS_2 sample by Raman spectra. The difference between the peak the in-plane peak (E^1_{2g}) and out-of-plane peak (A^1_{1g})

is 18.5 cm^{-1}, indicating the monolayer nature.

Supplementary Note 3. Monolayer WSe_2 FET characteristics

Supplementary Figure 2a shows the optical image of monolayer WSe_2 FETs used in this experiment, in which the monolayer sample is confirmed by the Raman spectra (Supplementary Figure 2b). The transfer characteristics of this device under $V_{D_1S_1} = 1$ V bias with temperature varying from 200 K to 6.1 K is presented in the Supplementary Figure 2c. The Edge conductance extracted according to the equations in the Supplemental Note 1 and the ratio of edge conductance to total conductance is demonstrated in Supplementary Figure 2d. The ratio of G_{edge}/G_{total} is increasing with lowering temperature, which is similar to the results in MoS_2 shown in Fig. 1d in the main text. With decreasing temperature, the carriers are freezing out in bulk of WSe_2, thus the bulk conductance reduces and the ratio increase. Therefore, as temperature lowering, carriers flow at the edge of monolayer WSe_2.

Supplementary Note 4. DFT calculations of monolayer MoS_2 edge states

First-principles calculations for electrical properties of edge states based on density functional theory (DFT) were conducted using the Perdew-Burke-Ernzerhof (PBE) version of the generalized gradient approximation (GGA). The calculated band structures of a 2D sheet crystal and a nanoribbon of the monolayer MoS_2 are illustrated in the Supplementary Figure 4a. In the case of the monolayer crystal, the band structure features an energy gap of 1.8 eV, which indicates a semiconductor property of the 2D sheet. However, in the case of the nanoribbon, it can be seen that additional bands (highlighted by the red line in the Supplementary Figure 4a) cross the Fermi level (highlighted by the horizontal black lines in the Supplementary Figure 4a), indicating the existence of metallic states. These additional states are mainly localized at the edge atoms as revealed by the charge density distribution of the nanoribbon as shown in the Supplementary Figure 4b, confirming that the metallic states are associated with the edge atoms. At this point, it should noted that the DFT calculations used for band structure only apply to the ribbon geometry and therein the need for a real space model.

Supplementary Note 5. Finite-element Analysis

We simulate the voltage variation of the MoS_2 sample in Fig.4e by COMSOL Multiphysics, in

which the sample contains two parts: one is bulk (highlighted by the violet) and another is edge (highlighted by the grey) as shown in the Supplementary Figure 5. The width of edge used in this simulation is 100 nm. The conductivity of the bulk part and edge part is 0.1 S/m and 10000 S/m based on the Reference [1]. The bias condition is same with the one in Figure 4d.

Supplementary Note 6. Evidence for non-Coulomb-blockade mechanism

Coulomb blockade transport occurs in a system with an array of small conductive islands connected by narrow junctions with larger tunneling resistance and small self-capacitance[2]. The current and voltage are expected to obey the power law relation as $I = \alpha(V - V_{\mathrm{T}})^{\xi}$, where α is the fitting parameter, V_T is the threshold voltage below which the current is suppressed and ξ is the power-law exponent. In this model, the threshold voltage (V_{T}) is linearly inversely proportional to temperature (T) as $V_T = V_T(0) \times (1 - \beta T)$. From our experimental data, we plotted the $(V_{\mathrm{T}} - T)$ curve under gate voltage of 60 V in the Supplementary Figure 6. The V_T increases with lowering temperature, but not in a linear relation. So our experimental data do not support a Coulomb blockade model.

We then tried to fit the experimental data in Fig. 1c in the main text with $I = \alpha(V - V_T)^{\xi}$ as shown in the set of Supplementary Figure 6, in which the solid lines are fits using the equation $I = \alpha(V - V_{\mathrm{T}})^{\xi}$ with α and ξ values of 0.95×10^{-6} and 1.4. However, the mismatch between fitting curves and measured data clearly shows that the *I*-*V* relation could not be fitted by the same power exponent (ξ) at different temperatures.

Also for Coulomb blockade model, it would be supposed that for an array of N disks on a sample, the elemental threshold voltage (V_{T}) per disk can be simply modelled by simple energy for an isolated island with a capacitance C_0: $V_{\mathrm{T}}(0)/N = e/2C_0$. We can roughly use the same typical value as reported in Reference 1: $C_0 = 6 \times 10^{-19}$ F, $e = 1.6 \times 10^{-19}$ C and $N_{\min} = 4$. So the elementary threshold voltage $V_{\mathrm{T}}(0)$ is estimated to be no smaller than 0.533 V. However, as shown in the Supplementary Figure 6, the elemental threshold voltage $V_{\mathrm{T}}(0)$ is estimated to be around 0.25 V, far from 0.533 V.

So as discussed above, the Coulomb blockade mechanism does not reflect the characteristics of charge transport in our monolayer MoS_2 FET.

Supplementary Note 7. Universal scaling of the current-voltage characteristics of monolayer WSe_2 FET

The output curve ($I_{DS} - V_{12}$) with various temperature measured in this monolayer WSe_2 FET presented in the Supplementary Figure 7a. The nonlinear current-voltage characteristics is clearly observed. According to the equation (1) in the main text, the I-V data is plotted $I_{DS}/T^{1+\alpha}$ versus qV_{12}/k_BT and this data can be scaled to a universal curve with α=2.1, β=3 and $\gamma^{-1} = 100$ as shown in the Supplementary Figure 7b. This is solid evidence for Luttinger liquid.

Supplementary Note 8. Scaling of the current-voltage characteristics of monolayer MoS_2 FET

In order to investigate the relationship between scale currents ($I/T^{1+\alpha}$) and edge length (L_{edge}), we further investigate the MoS_2 devices with different edge lengths, L_{edge}=3.06 µm, 4.76 µm and 8.5 µm. The scaled currents $I/T^{1+\alpha}$ of monolayer MoS_2 with different channel lengths (L_{edge}) are presented as functions of qV/k_BT in the Supplementary Figure 8a with α equal to 2.2. For each FET, all measurements for different voltages and temperatures collapse onto a single curve. The parameter α does not depend on the channel length, but is related only to the different sample materials. The position of crossover between the linear and the super-linear region are shown by the dotted line in the Supplementary Figure 8a. This position of crossover, determined by the parameter γ^{-1} in the equation (1) as described in the main text, increases with the edge states' length, which is shown in the Supplementary Figure 8b. The edge length increases with channel length, so the number of tunneling barriers also increase.

Supplementary Note 9. Discussion of parameters in 1D Luttinger liquid theory

In Luttinger liquid, the strength of the interaction between electrons (holes) is described by the parameter g. For non-interacting electron (hole) gas $g = 1$ while for strong repulsive interactions $g < 1$. The tunneling exponent α can be related to parameter g by the following equation[3]:

$$\alpha = (g^{-1} - 1)/4 \qquad (5)$$

This equation for end contacts is applicable here because the two middle electrodes in the four-probe configuration of the current measurement essentially make end contacts with Luttinger liquid.

Here, based on the fitting parameter, $g = 0.102$ for $\alpha = 2.2$ in MoS_2 FET and $g = 0.106$ for $\alpha = 2.1$ in WSe_2 FET can be obtained, which means the electron interaction is a strong repulsive interaction in edge-state-dominated MoS_2 and WSe_2 crystals and this result can be generally extended to all TMD crystals.

For a screened Coulomb interaction, the tunneling exponent α is related to the Coulomb energy U and the Fermi energy E_F by the following expression[4]:

$$2\alpha = U/2E_F \quad (6)$$

in which, the Coulomb energy

$$U = (e^2/4\pi\varepsilon)n_{1D} \quad (7)$$

(where $n_{1D} = 2\,k_F/\pi = 2mv_F/\pi\hbar$ is the one-dimensional electron charge density in edge states), and the Fermi energy can by calculated by the following equation

$$E_F = \frac{1}{2}m^* v_F^2 \quad (8)$$

(where m^* is the effective mass[5] of MoS_2 and v_F is the Fermi velocity).

Combining Supplementary Equations (6), (7) and (8), the Fermi velocity is expressed by the following equation:

$$v_F = e^2/2\pi\varepsilon h\alpha = 1.1441 \times 10^5\ m/s, \quad (9)$$

where e is the elementary electron charge, ε is the dielectric constant[6] of monolayer MoS_2 and h is Planck's constant. The Fermi velocity of MoS_2 based on one-dimension edge states has the same magnitude with the one calculated by other method[7], $5.3 \times 10^5\ m/s$.

Supplementary Note 10. Summary of all MoS_2 and WSe_2 devices.

All devices are summarized in Supplementary Figure 9, Supplementary Figure 10 and Supplementary Figure 11. They all show that the Luttinger liquid behavior dominates the charge transport of TMD materials at temperature below 50 K as shown in MoS_2 devices (Supplementary Figure 9) and WSe_2 devices (Supplementary Figure 10). The extracted parameters for MoS_2 and WSe_2 are summarized in the Supplementary Table 1.

Supplementary Note 11. Schematic of Luttinger-liquid-based charge transport on monolayer MoS_2 edge states.

A schematic process of edge-state-dominated 1D metallic charge transport in atomic-thin 2D

crystal based on Luttinger liquid model is drawn in the Supplementary Figure 11. It shows that electrons transport from one Luttinger liquid to another Luttinger liquid through 1D tunneling across possible defects or impurities along the metallic edges in monolayer 2D materials, as shown above by the example of MoS_2 crystal.

Supplementary Note 12. Temperature-dependence Resistance of Monolayer MoS_2.

Peierls transition have seen observed in many 1D metallic systems, in which the periodic lattice deformation and change the boundary of Brillouin zone to reduce the system energy by opening a small bandgap at the Fermi level. In addition, the Peierls transition is associated with a metal-to-insulator transition due to the bandgap opening and thus a sudden increase in sample resistance at the transition temperature. Thus, we investigate temperature-dependent resistance relationship in our sample as shown in the Supplementary Figure 12. The resistances clearly increase with lowering temperature at the constant current of 1 μA, 100 nA and 27 nA. However, all the temperature-dependency resistances do not show sudden transition, which is a direct evidence that a Peierls transition would not occur in metallic edge states of TMD. On the other hand, the reference [8] investigated the metallic edge of MoS_2 by STM with atom resolution and exhibit a variation with a period two at the Mo edge, which, however, does not induce any change in the atomic or the electronic structure, contradictory to the Peierls transition. Based on the stated above, we believed that the likelihood of Peierls transition is low in metallic edge states of TMD.

Supplementary References:

1. Wu, D., Li, X., Luan, L., Wu, X., Li, W., Yogeesh, M. N. *et al.* Uncovering edge states and electrical inhomogeneity in MoS_2 field-effect transistors. *Proc. Natl. Acad. Sci. U.S.A.* 113, 8583-8588 (2016).
2. Akai-Kasaya, M., Okuaki, Y., Nagano, S., Mitani, T., Kuwahara, Y. Coulomb blockade in a two-dimensional conductive polymer monolayer. *Phys. Rev. Lett.* 115, 196801 (2015).
3. Bockrath, M., Cobden, D. H., Lu, J., Rinzler, A. G., Smalley, R. E., Balents, L. *et al.* Luttinger-liquid behaviour in carbon nanotubes. *Nature* 397, 598-601 (1999).
4. Levy, E., Sternfeld, I., Eshkol, M., Karpovski, M., Dwir, B., Rudra, A. *et al.* Experimental evidence for Luttinger liquid behavior in sufficiently long GaAs V-groove quantum wires. *Phys. Rev. B* 85, 045315 (2012).
5. Yoon, Y., Ganapathi, K., Salahuddin, S. How good can monolayer MoS_2 transistors be? *Nano Lett.* 11, 3768-3773 (2011).
6. Cheiwchanchamnangij, T., Lambrecht, W. R. L. Quasiparticle band structure calculation of monolayer, bilayer, and bulk MoS_2. *Phys. Rev. B* 85, 205302 (2012).
7. Li, X., Zhang, F., Niu, Q. Unconventional quantum hall effect and tunable spin hall effect in Dirac materials: application to an isolated MoS_2 trilayer. *Phys. Rev. Lett.* 110, 066803 (2013).
8. Bollinger, M. V., Lauritsen, J. V., Jacobsen, K. W., Norskov, J. K., Helveg, S., Besenbacher, F. One-dimensional metallic edge states in MoS_2. *Phys. Rev. Lett.* 87, 19683 (2001).
